# Genomic sequences and annotations of two *Pseudomonas* species isolated from marine and terrestrial habitats

**DOI:** 10.1128/mra.00373-24

**Published:** 2024-08-27

**Authors:** Eric Manirakiza, Timothée Chaumier, Leïla Tirichine

**Affiliations:** 1 Nantes Université, CNRS, US2B, UMR 6286, Nantes, France; 2 Institute for Marine and Antarctic Studies (IMAS), Ecology and Biodiversity Centre, University of Tasmania, Hobart, Tasmania, Australia; California State University San Marcos, San Marcos, California, USA

**Keywords:** *Pseudomonas stutzeri*, *Pseudomonas chlororaphis*, environmental microbiology, bacterial genomics

## Abstract

Here, we present the complete genome sequences and annotations of two species of the *Pseudomonas* genus isolated from marine and terrestrial environments. Both genomes and their annotations are available on BacBrowse (https://BacBrowse.univ-nantes.fr). This study will contribute to a better understanding of the diversity present within the *Pseudomonas* genus.

## ANNOUNCEMENT

*Pseudomonas* is one the most diverse bacterial genera, is widely distributed in nature, and is the genus with the highest number of species among the gram-negative bacteria (1). Members of this genus show remarkable metabolic and physiological flexibility, enabling them to thrive in diverse terrestrial and aquatic environments. They are of great interest because of their importance in both plant and human diseases as well as their expanding potential in biotechnological applications (2–4). The *Pseudomonas* species used in this study are *Pseudomonas chlororaphis* (NCIMB 15121), isolated on 12 October 2013, from soil in Denmark, and *Pseudomonas stutzeri* (NCIMB 885) (5), isolated in 1930 from seawater in Loch Striven off the west coast of Scotland at a depth of 36 m. Upon isolation, *P. stutzeri* was plated on nutrient agar (6), and *P. chlororaphis* was plated on a Luria–Bertani (LB) medium (7). Both strains were retrieved from −80°C frozen stocks and plated on a solid LB medium, and individual colonies were selected for culturing in liquid LB at 30°C with shaking at 180 rpm for 48 hours. After cell harvest, genomic DNA was extracted using the Promega Wizard HMW DNA Extraction Kit (A2920) according to the manufacturer’s instructions. The DNA libraries were prepared using SMRTbell Express Template Prep Kit 2.0 following the manufacturer’s instructions. A total of 4 µg of genomic DNA was sheared with Covaris g-TUBES at 4,000 rpm, resulting in fragment sizes of 11,290 and 10,730 bp for *P. stutzeri* and *P. chlororaphis*, respectively. DNA was sequenced by Genome Quebec using the Pacific Biosciences Sequel platform with circular consensus sequencing (CCS). CCS reads were generated from subreads using CCS 6.4.0 and assembled with Canu (v2.2) (8), which determined that both genomes are circular. The completeness and contamination levels of the assemblies were estimated using CheckM (v1.2.1) with the bacterial lineage workflow (9). The assembled genomes were then annotated using anvi’o v7.1 (10), which runs NCBI’s Clusters of Orthologous Groups database (COG20) to assign functions to genes. This revealed 5,923 and 4,295 coding regions for *P. chlororaphis* and *P. stutzeri,* respectively. Additional gene annotations were conducted with DFAST 1.2.20 (11), Prokka (12) with a compliant parameter (v1.14.6), and EggNOG (v2.1.12) (13). The parameters -m [diamond] and pfam_realign [realign] were used for EggNOG. Genome features for both strains are summarized in Table 1.

**TABLE 1:** Genome features of *P. chlororaphis* and *P. stutzeri*^*a*^

	*P. chlororaphis*	*P. stutzeri*
Number of subreads	962,522	833,208
Number of CCS reads	49,060	45,998
Average length of CCS reads (bp)	6,979.45	7,087.93
N50 of CCS reads (bp)	9,501	9,320
Number of contigs	1	1
Mean genome coverage	51.5×	69.8×
Genome size (bp)	6,675,668	4,639,068
Contamination	0	0
Completeness (%)	98.28	100
GC content (%)	63	61.3
Coding region (%)	88.3	89.5
Average length CR (bp)	995.51	966.71
IR (%)	11.9	10.2
Average length IR (bp)	164.90	147.05
Total repeats	461	218
Total tRNAs	73	63
Total rRNAs	16	12
Total pseudogenes	793	358

^*a*^ CR, coding region; IR, intergenic region. GC content was determined using Quast (14).

Using AntiSMASH (7.1.0) (15), the prediction of secondary metabolites revealed shared biosynthetic gene clusters (BGCs) for NRPS, RiPP, redox cofactor, NAGGN, and betalactone in both species. Specific BGCs including hydrogen cyanide, hserlactone, NI-siderophore, and arylpolyene were exclusively identified in *P. chlororaphis*, whereas *P. stutzeri* specifically contained BGCs for terpene production. These differences may result from their contrasting terrestrial and aquatic habitats, reflecting variations in environmental conditions and microbial competitors. Both genomes are accessible on BacBrowse (https://BacBrowse.univ-nantes.fr), enabling the exploration of *Pseudomonas* genomes and bioactive compound identification. Identified genes and pathways can be genetically modified in tractable species like those in this study.

## Data Availability

The genome sequences of *P. chlororaphis* and *P. stutzeri* have been deposited at the NCBI under assemblies ASM3849947v1 and ASM3850004v1, respectively (BioProject PRJNA1101843). The genome sequencing raw reads have been deposited in the NCBI Sequence Read Archive under accession numbers SRR28730576 and SRR28730575, respectively. The annotations are available on Zenodo under the following DOI: https://zenodo.org/doi/10.5281/zenodo.11657795.
